# Effect of Low-Molecular-Weight Organic Acids on Migration Characteristics of Pb in Reclaimed Soil

**DOI:** 10.3389/fchem.2022.934949

**Published:** 2022-07-14

**Authors:** Yonghong Zheng, Yating Li, Zhiguo Zhang, Yuning Tan, Weiqing Cai, Chengnan Ma, Fangling Chen, Jiangwei Lu

**Affiliations:** ^1^ School of Earth and Environment, Anhui University of Science and Technology, Huainan, China; ^2^ Anhui Engineering Laboratory for Comprehensive Utilization of Water and Soil Resources and Ecological Protection in Mining Area with High Groundwater Level, Huainan, China; ^3^ National Engineering Laboratory for Protection of Colliery Eco-environment, Huainan, China; ^4^ Hefei Comprehensive National Science Center, Institute of Energy, Hefei, China

**Keywords:** heavy metal, plumbum, citric acid, malic acid, form, pollution remediation

## Abstract

The effect of low-molecular-weight organic acids (citric acid and malic acid) on the migration characteristics of Pb in contaminated soils was explored in this study. Reclaimed soil was collected from the coal gangue hill area of the Panyi mine in Huainan City (China). The effect of citric acid and malic acid on the form of Pb present in the reclaimed soil was analyzed by spiking soil samples and simulating Pb-contaminated soil. The results indicate the following. 1) With increased concentration of exogenous Pb, the activity of Pb in the reclaimed soil was effectively improved. 2) The addition of citric acid and malic acid both resulted in an increased fraction of exchangeable Pb in the soil, which effectively promoted the active Pb fraction. As the concentrations of citric acid and malic acid increased, the active Pb fraction of the reclaimed soil increased accordingly. The Pb activation effect of citric acid was observed to be greater than that of malic acid. 3) With extended soil aging time, the activation effect of organic acids on Pb weakened, with the loosely bound Pb gradually transforming into strongly bound Pb. Chelating agents can activate heavy metals in soil, mainly through the combination of chelating agents and heavy metal ions in the soil solution to form soluble metal chelates, so as to increase the bioavailability of heavy metals in soil to plant roots. Therefore, adding citric acid can be considered as a strategy to enhance the efficiency of reclaimed soil remediation because of the ability of Pb activation.

## 1 Introduction

In recent years, the environmental impacts of anthropogenic activities have become increasingly serious because of rapid urbanization and industrial and agricultural development. Lead (Pb) fraction in the soil environment exceeded natural threshold and seriously affected the soil ecology. Pb can be ingested by human through the food chain, thereby negatively affecting health (Zhang et al., 2021). According to the National Soil Pollution Survey Report in 2014, the main contaminants of arable land were heavy metals including cadmium (Cd), nickel (Ni), and Pb. Among them, the ratio of exceedance of Pb in soil reached 1.5%, while the ratio of heavily contaminated points of Pb was 0.1% (Wang et al., 2014). Soil Pb contamination can be caused by Pb mining activities (Zhang et al., 2012; Foulds et al., 2014), Pb-containing wastewater discharged during industrial production (Zheng and Zhang, 2017), excessive use of chemical fertilizers and pesticides (Pichtel et al., 2000), and use of Pb batteries (Picard et al., 2017). Among them, heavy metal-containing exhaust gas deposition, wastewater irrigation emitted by metal mining, and dissolution and diffusion of solid wastes soil are the main ways of heavy metal pollution (Zhou et al., 2014). Mining can result in a large accumulation of heavy metals in the surrounding and downstream rivers and soils. Coal gangue is the largest source of industrial solid waste in China. Moreover, the long-term exposure of coal gangue to sun and rain can increase the soil pollution owing to the release of heavy metal elements (Shang et al., 2021). Some of the reclaimed area soils are affected by coal mining and reclamation filling activities with increased Pb fraction (Zheng et al., 2013).

In the past few years, the soil remediation industry regarding heavy metal has developed rapidly in China, with a significant increase in the number of projects and continuous innovation. As a chemical remediation strategy, organic complexation is relatively mature, low cost, and fast acting; however, secondary pollution of organic complexation has impeded widespread adoption of this technology (Liu GH et al., 2018). Engineering measures, often taken for physical remediation, cost a lot of material and financial resources, and the construction process is prone to cause serious fluctuations in the soil and damage the soil structure (Tomohito et al., 2010). Microbial remediation has limited fixation of heavy metals and poor metabolic capacity (Jin et al., 2018). Compared with other remediation technologies, phytoremediation is a more green and environmentally friendly remediation technology. Phytoremediation is a sustainable *in situ* soil treatment technology (Amanullah et al., 2016) and can be applied in combination with other remediation technologies to significantly improve the performance of heavy metal pollution treatment (Khalid et al., 2016). Phytoremediation is a technical method to remove heavy metals from polluted soil by using the physical and chemical action of plants, which can effectively reduce secondary pollution and improve the physical, chemical, and biological environment of polluted soil. Plant-enhanced remediation technology can obtain higher remediation efficiency by increasing plant biomass or increasing the content of heavy metals in plants. Furthermore, chelating agents can change the growth state, biochemical characteristics, and resistance mechanism of plants and promote the absorption and enrichment of heavy metals in polluted soil. Therefore, plant chelator–combined remediation technology is widely used in the plant extraction and enhancement process of heavy metal-contaminated soil and has broad application prospects (Komarek et al., 2007). Natural chelating agents mainly refer to low-molecular-weight organic acids, such as citric acid, tartaric acid, and oxalic acid. They are biodegradable and environment-friendly and are considered to have strong application potential in the phytoremediation of heavy metal-contaminated soil (Chen et al., 2020). For instance, Huang et al. (2008) studied the removal effect of citric acid, oxalic acid, and acetic acid on heavy metals such as CD and Pb in sludge and analyzed the morphological changes and bioavailability of heavy metals in sludge. Their results showed that the removal rate of heavy metals in sludge increased with the increase of reaction time and acid concentration, and the concentration of exchangeable heavy metals increased in varying degrees. Inge and Hans (2000) investigated the effects of citric acid and arginine on the adsorption of heavy metal nickel in soil at 0.5 and 5 mmol/L. Their results showed that both of citric acid and arginine combined with nickel resulted in the formation of metal complexes. Yang and Liao (2010) used citric acid, oxalic acid, and tartaric acid to leach heavy metals to pollute farmland soil. Results showed that the three acids had good extraction effects on heavy metals such as Cd, Pb, copper, and zinc in soil. The effective components of heavy metals (Li et al., 2017) can react with the substances in the soil, and the reaction products with different form can change the physicochemical properties of soil and subsequently affect plant growth. Therefore, it is crucial to understand the form of the reaction products for the heavy metal remediation of soil (Abhishek et al., 2018). Organic acid is a natural chelating agent that can be electrically adsorbed, complexed, and chelated with heavy metals to improve their bioeffectiveness, which is very helpful in remediation of soil heavy metal pollution (Li et al., 2017; Ye et al., 2018).

In the present study, the Pb soil contamination was simulated using reclaimed soil from the mining area of Huainan City (China). The effect of exogenous heavy metals and endogenous organic acids on the form, morphology, and migration of Pb in reclaimed soil was investigated. The methodology and outcomes of the present study provide insights on the reclamation and management of heavy metal pollution in mining area.

## 2 Materials and Methods

### 2.1 Sample Collection and Processing

The soil used for analysis was collected from the surface layer (0–20 cm depth) of reclaimed soil in the Panyi mine reclamation area of Huainan City, China. The soil samples were spread evenly in an air-drying tray, and then biological matter, debris, gravel, and other impurities were removed before the soil samples were placed in a well-ventilated place to dry naturally without exposure to direct sunlight. The air-dried soil samples were ground and passed through an 18-mesh sieve and then stored for later use.

### 2.2 Experimental Procedure

#### 2.2.1 Analytical Methods and Instruments

The basic physical and chemical properties of soil were determined according to the standard methods specified for standardized soil analysis technology method (Zha, 2017). Allosteric Pb in reclaimed soils was determined by triple acid wet digestion (HF-HNO_3_-HClO_4_). A modified version of the Tessier five-step continuous extraction method (Qian et al., 2011) was used to extract the different forms of Pb present in contaminated soil, including the following: exchangeable (F1), carbonate bound (F2), iron and manganese oxide bound (F3), organic matter bound (F4), and residual (F5). The extraction process is shown in Table 1. The Pb fraction in the test solution was measured via inductively coupled plasma mass spectrometry (ICP-MS; PE NexION 300).

**TABLE 1:** Tessier continuous extraction method.

Heavy metal form	Extractant	Operating conditions
1. Exchangeable	8 ml 1 mol/L MgCl_2_ (pH = 7.0)	Vibrate for 1 h at room temperature
2. Bound to Carbonate	16 ml 1 mol/L NaAc (pH = 5.0)	Shake at room temperature for 5 h
3. Bound to Iron and Manganese Oxide	16 ml 0.04 mol/L	(96 ± 3) °C water bath intermittently shake for 6 h
	NH_2_OH·HCI (25% HAc)	
4. Bound to Organic Matter	3 ml 0.01 mol/L HNO_3_	(85 ± 2)°C water bath extraction for 5 h, and finally add NH_4_Ac to prevent re-adsorption and shake for 30 min
	5 ml 30%H_2_O_2_(pH = 2)	
5. Residual	HF-HNO_3_-HClO_4_	Completely dissolve

#### 2.2.2 Preparation of Pb-Contaminated Soil

According to the Chinese National Soil Environmental Quality Agricultural Land Soil Pollution Risk Control Standards (GB 15618-2018), five pollution concentration gradients were applied. First, different concentrations of Pb(NO_3_)_2_(GR) aqueous solution were added to the soil samples, resulting in Pb concentrations in the simulated contaminated soil of 0, 100, 200, 400, and 800 mg/kg, with the water-holding capacity maintained at 40–60%. Simulated samples were placed in an artificial climate incubator (RCX-180F) in simulated sunlight and maintained for 7 days at 25°C; then, the final Pb-contaminated soil was air-dried.

#### 2.2.3 Organic Acid Addition Experiment

First, the effect of low-molecular-weight organic acid type and concentration on the Pb form in soil was assessed by adding different concentrations of citric acid and malic acid to the prepared soil samples, as shown in Table 2. The samples were collected after 7 days of incubation at 25°C and then air-dried and sieved. All samples were prepared in triplicate.

**TABLE 2:** Concentration of organic acids added.

Types of organic acids	Molecular formula	Molecular weight	Add concentration (mmol/L)		
Citric acid	C_6_H_8_O_7_	192.14	0	1	10
Malic acid	C_4_H_6_O_5_	134.09	0	1	10

Second, the effect of low-molecular-weight organic acids on the form of Pb in soil was assessed under different incubation duration conditions. A 400 mg/kg sample of 20 g Pb-contaminated soil was combined with 12 ml of 10 mmol/L citric acid or malic acid, as shown in Table 2. Spiked soils were incubated for 30 days at 25°C and then air-dried and sieved before being preserved, with samples collected at intervals of 1, 3, 5, 7, 15, and 30 days. The collected soil samples taken out are air-dried and sieved. All samples were prepared in triplicate.

### 2.3 Quality Control and Data Processing Methods

The acids used were all of superior purity, and other chemical reagents were all of analytical purity. The heavy metal (Pb) standard stock solution was prepared from plumbum nitrate (Pb(NO_3_)_2_)(GR) (1,000 mg/L).

The relative deviations were between −2.12 and 2.28% for the calibration of all-state Pb fraction in the simulated Pb-contaminated soil, as shown in Table 3.

**TABLE 3:** Fraction and deviation of Pb in the simulated soil.

Measurement objects	Simulation of the predicted concentration of Pb-contaminated soil (mg/kg)	Measured mean all-state Pb fraction (mg/kg)	Relative deviation (%)
A	100	102.03	2.03
B	200	197.70	−1.15
C	400	409.11	2.28
D	800	783.04	−2.12

The data obtained from experiments were organized in Excel 2019, and graphs were prepared using Origin2021 software.

## 3 Results and Analysis

### 3.1 Physicochemical Properties of Soils in Coal Mine Reclamation Areas

Analyzing the data in Table 4 and Table 5, and comparing them with the nutrient grading standard of the second national soil census (Guo et al., 2020), it can be seen that the average pH value of the soil in the reclaimed area of Panyi mine is 7.80, which is alkaline. The mean value of soil bulk weight is 1.33 g/cm^3^, which is on a too tight level. The average value of organic matter fraction is 4.13 g/kg, which is at the level of 6 (extreme deficiency). The average value of quick-acting potassium fraction was 191.03 mg/kg, suggesting a high level of grade 2. The average value of effective phosphorus fraction was 10.41 mg/kg, which is in the middle to upper level of 3. The average value of alkaline nitrogen fraction is 28.98 mg/kg, which is a very low level of grade 6. The soil in the reclamation area is affected by artificial filling and reclamation activities. Soil in the reclamation area was affected by artificial filling and reclamation activities, which disturbed the original soil sequence, and mechanical rolling caused by engineering construction, resulting in tight surface soil bulk density, and the soil nutrients, except for the high fraction of fast-acting potassium and effective phosphorus, are all at very low levels.

**TABLE 4:** Nutrient classification standard of the second national nutrient census.

Level	Organic matter (g/kg)	Quick-acting potassium (mg/kg)	Available phosphorus (mg/kg)	Hydrolyzable nitrogen (mg/kg)
Level 1	>40	>200	>40	>150
Level 2	30–40	150–200	20–40	120–150
Level 3	20–30	100–150	10–20	90–120
Level 4	10–20	50–100	5–10	60–90
Level 5	6–10	30–50	3–5	30–60
Level 6	<6	<30	<3	<30

**TABLE 5:** Physicochemical properties of soils in reclaimed areas.

Capacity (g/cm^3^)	pH	Organic matter (g/kg)	Quick-acting potassium (mg/kg)	Available phosphorus (mg/kg)	Hydrolyzable nitrogen (mg/kg)
1.33	7.80	4.13	191.03	10.41	28.98

### 3.2 Pb Form Distribution in Soil Contaminated by Exogenous Pb

**FIGURE 1:**
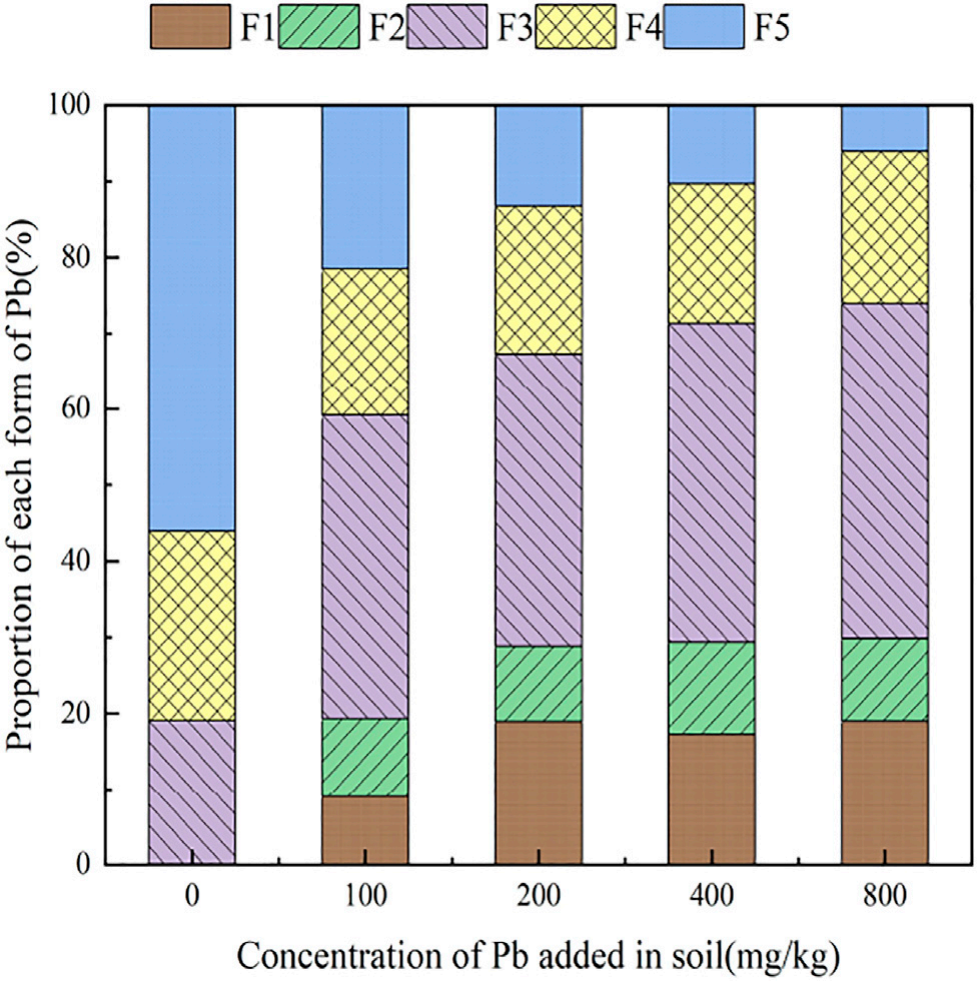
The distribution of Pb form in the soil after adding exogenous Pb.

The form distribution pattern of Pb in soil contaminated with increasing concentrations of exogenous Pb, is shown in Figure 1. It can be seen from Figure 1 that the main form of Pb in the original soil sample (prior to the addition of exogenous Pb) is the residual-state Pb, accounting for 56.08% of the total Pb concentration. The second most abundant form in the original sample was the organic-combined state, which accounts for 24.88% of the total Pb concentration, with the remaining fraction being in an exchangeable state (0.00%), carbonate-combined state (0.11%), and iron–manganese oxide-combined state (18.93%). With the addition of exogenous Pb, the percent fraction of Pb form in the soil has changed, and the fraction of Pb in the iron–manganese oxide-combined state gradually increased from 18.93% in the original soil to 44.13%, becoming the main form of Pb in the soil. This is because of the fact that exogenous Pb entered the soil and adsorbed to the surface of soil particles, and it gradually transformed to stable forms such as the iron–manganese oxide-combined state as the contact time with soil was prolonged. The exchangeable and carbonate-combined Pb first increased slightly and then remained unchanged, and the fraction of organic-bound Pb remained relatively unchanged. With the increase of exogenous Pb concentration, the residual-state Pb fraction gradually decreased from 56.08 to 6.04% in the original soil. This is because of the fact that the residual-state Pb is chemically stable, its formation process is calculated by geological age, and its mass fraction is not easily affected by external environmental changes, with the percentage of Pb fraction decreasing when the total amount of Pb increases. It can be seen that the addition of different concentrations of exogenous Pb to the soil can significantly change the distribution of each form of Pb in the original soil, which indicates that the form of Pb in the soil is equally affected by the total amount of Pb and the concentration of exogenous Pb.

### 3.3 The Influence of Organic Acids on the Form of Pb in Soil

#### 3.3.1 The Effect of Citric Acid on the Form of Pb in Soil

**FIGURE 2:**
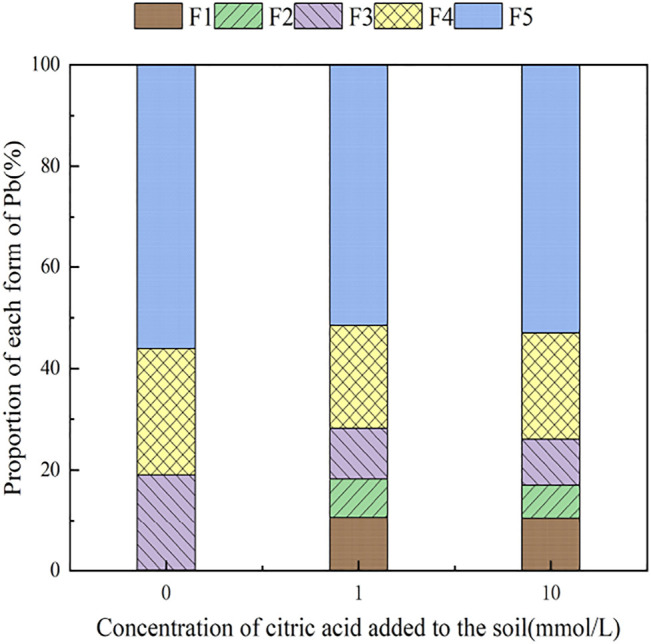
Distribution of Pb in the original soil after adding citric acid.

With the addition of different concentrations of citric acid, changes were observed in the distribution of Pb in the soil, as shown in Figure 2. As the concentration of citric acid increased, the fraction of exchangeable Pb and carbonate-bound Pb gradually increased, with their relative proportion in soil increasing from 0.00 and 0.11% to 0.49 and 6.5%, respectively. The proportion of iron–manganese oxide-combined state and organic-combined state Pb decreased gradually from 18.93 and 24.88% to 9.09 and 20.92%, respectively. The residual state remained largely unchanged owing to its high stability in the environment.

**FIGURE 3:**
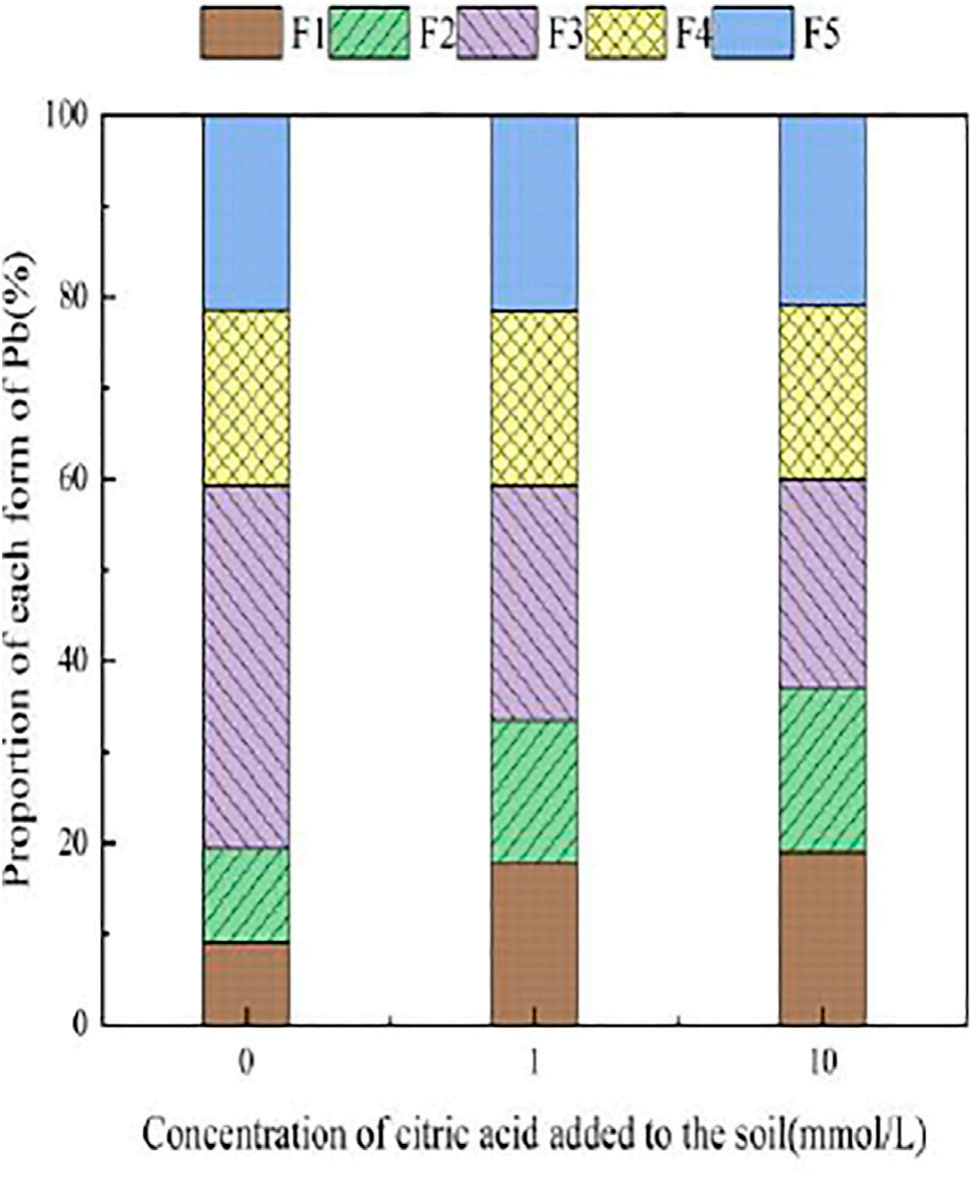
Distribution of Pb form in Pb-100.

**FIGURE 4:**
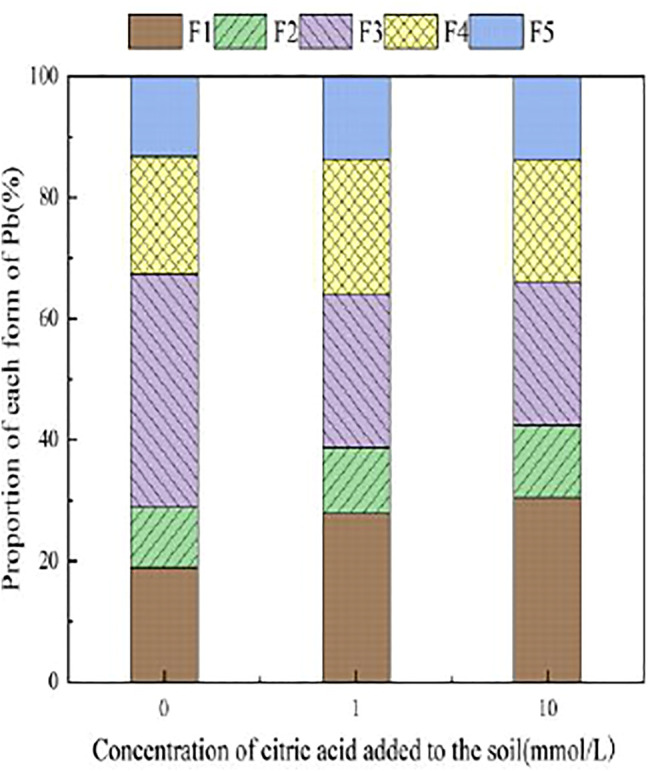
Distribution of Pb form in soil after adding citric acid and Pb-200 soil after adding citric acid.

**FIGURE 5:**
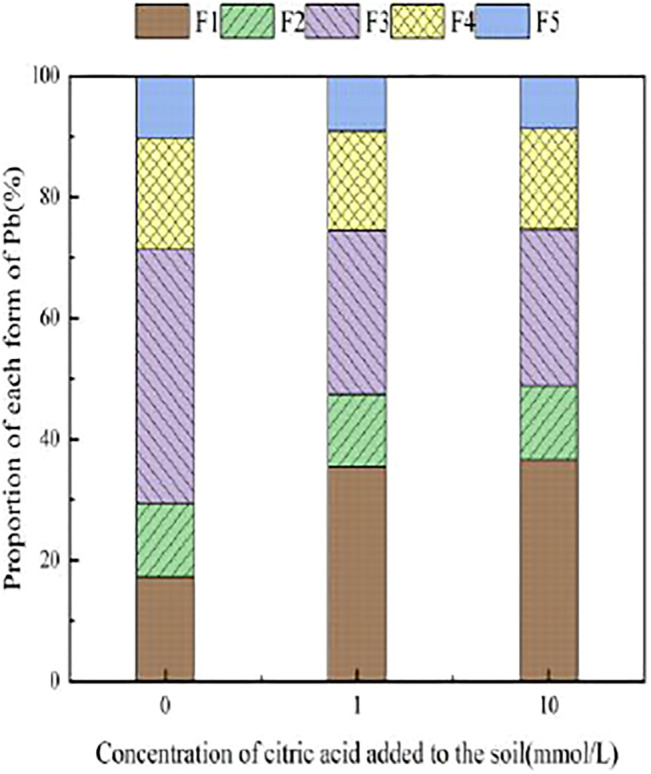
Distribution of Pb form in Pb-400.

**FIGURE 6:**
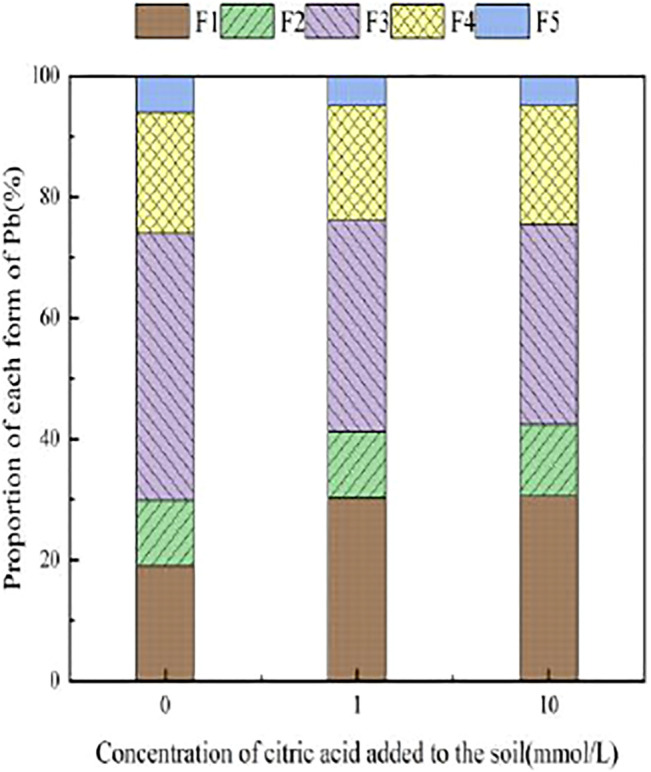
Distribution of Pb form in soil after adding citric acid and Pb-800 soil after adding citric acid.

Figures 3–6 illustrated the changes in the form of Pb in contaminated soil with the addition of different concentrations of citric acid. It can be seen that as the concentration of citric acid increased, the exchangeable Pb fraction gradually increased. The effect was especially visible in the 400 mg/kg Pb-contaminated soil, in which the exchangeable Pb fraction increased from 17.22 to 36.57%. The addition of citric acid to the soil was beneficial to increase the solubility and mobility of heavy metals in the soil. The carbonate-bound state Pb fraction did not change significantly and showed a slight increasing trend. The addition of 10 mmol/L citric acid to 400 mg/kg Pb-contaminated soil decreased the iron–manganese oxide-combined Pb fraction from 40.02 to 25.80%, which is because of the fact that the iron–manganese oxide-combined Pb fraction is susceptible to changes in soil pH. pH affects the mobility and biological effectiveness of heavy metals in soil. The addition of organic acids led to a decrease in soil pH, which increased the solubility of Pb and contributed to the conversion of the iron–manganese oxide-bound state to the exchangeable state, increasing its effectiveness (Zhang et al., 2008). The organic-bound Pb and residue Pb fraction did not change significantly because the organic-bound state is generally released only under strong oxidation conditions, while the residual state is generally present in the soil lattice of silicates and primary and secondary minerals, which are chemically stable and not easily affected by the external environment (Zheng et al., 2022).

#### 3.3.2 The Effect of Malic Acid on the Form of Pb in Soil

**FIGURE 7:**
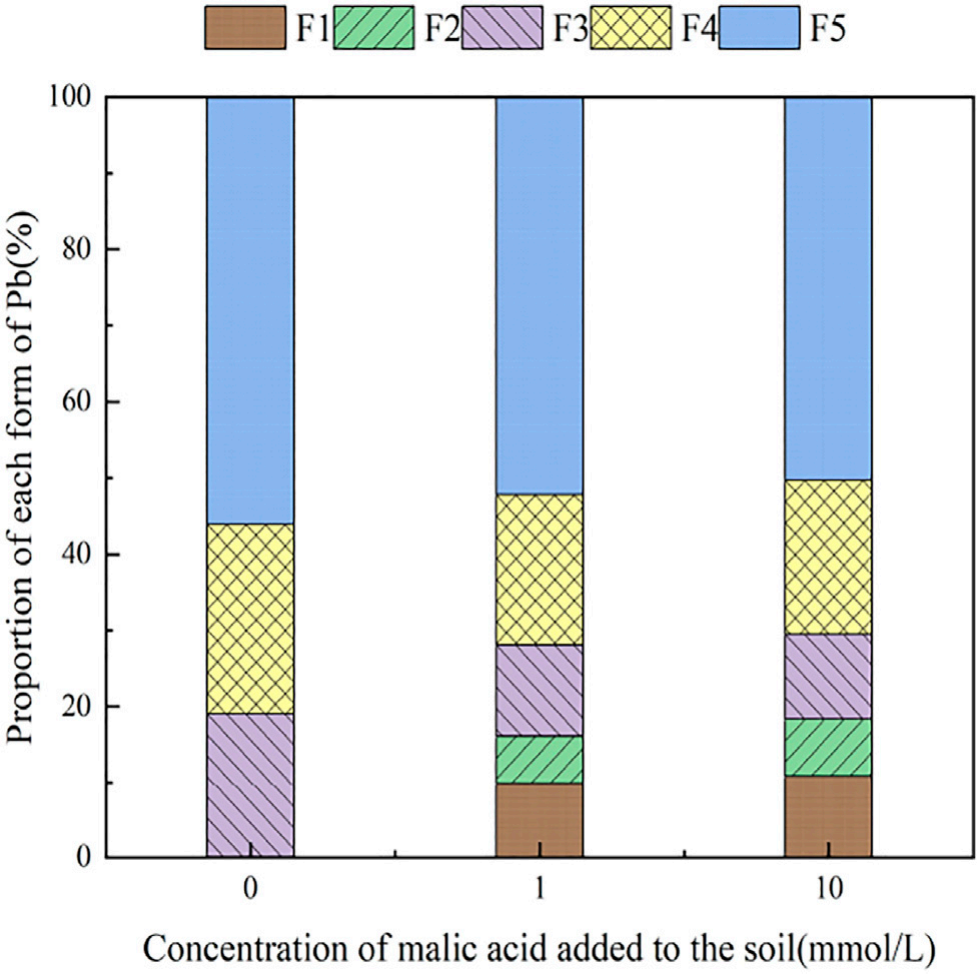
The distribution of Pb form in the original soil after adding malic acid.

Figure 7 presents the changes in Pb form with the addition of different concentrations of malic acid to soil. As shown, the addition of malic acid induced a downward trend in the proportion of iron–manganese oxide-bound Pb and organic-bound Pb, from 18.93 and 24.88% to 11.14 and 20.18%, respectively, with the observed change being approximately proportional to that induced by citric acid. The exchangeable Pb and carbonate-bound Pb contents gradually increased with increasing organic acid concentration, from 0.00 and 0.11% to 10.87 and 7.5%, respectively, with relatively similar increases compared to citric acid. The change in the residual-state Pb fraction was not significant.

**FIGURE 8:**
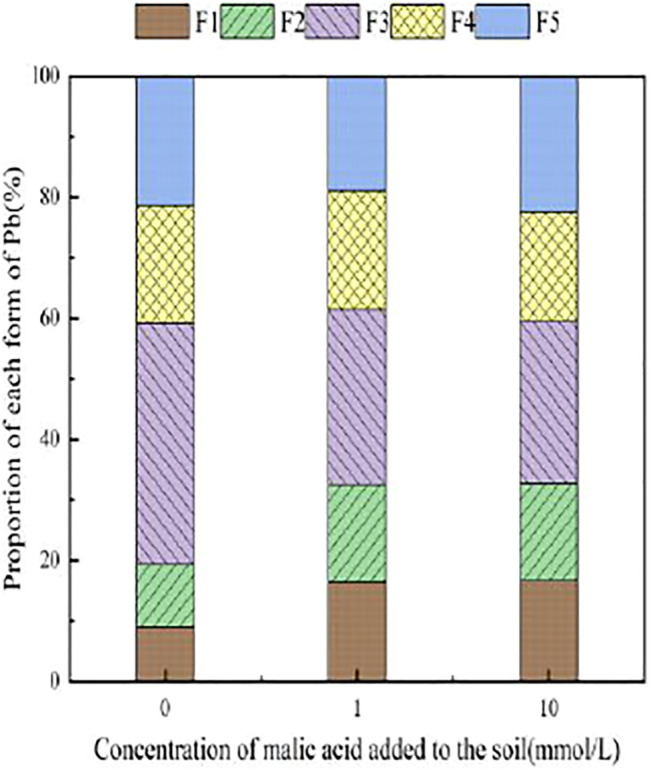
Distribution of Pb form in Pb-100.

**FIGURE 9:**
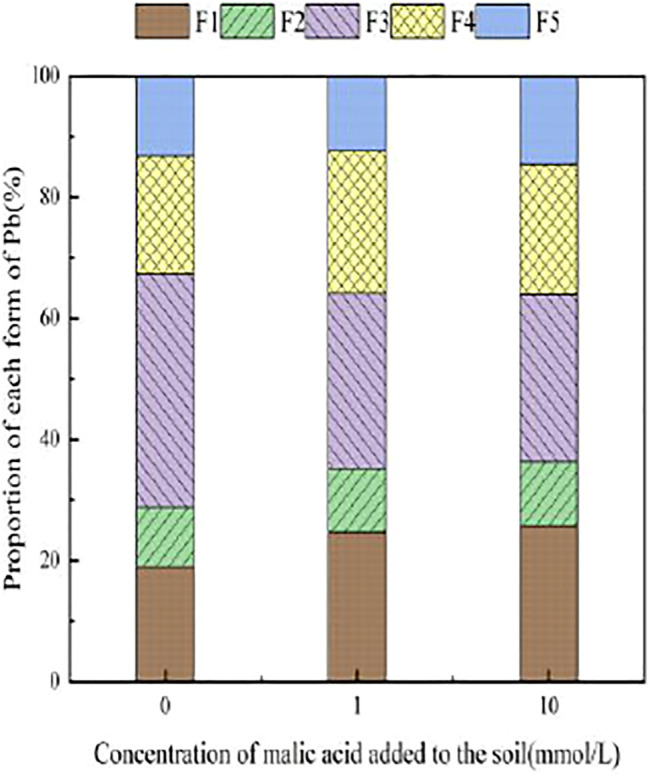
Distribution of Pb form in soil after adding malic acid and Pb-200 soil after adding malic acid.

**FIGURE 10:**
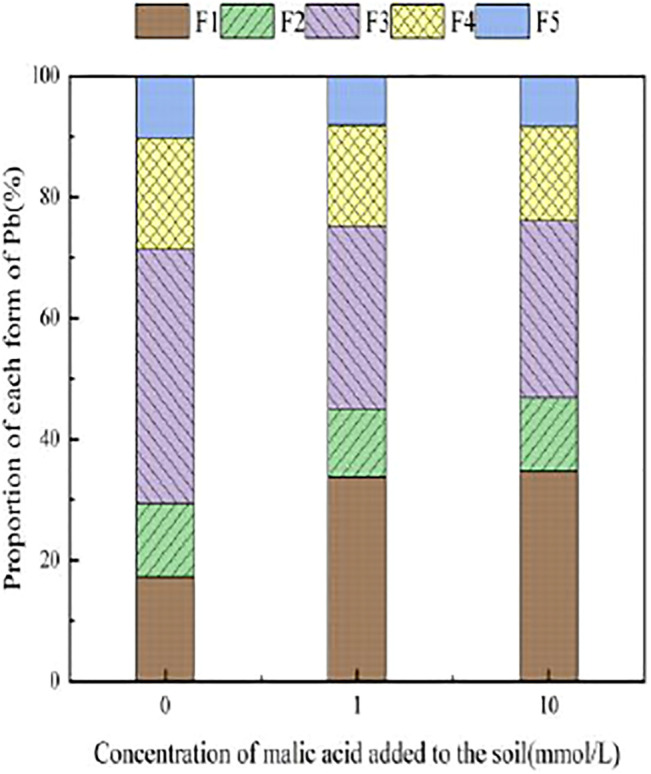
Distribution of Pb form in Pb-400.

**FIGURE 11:**
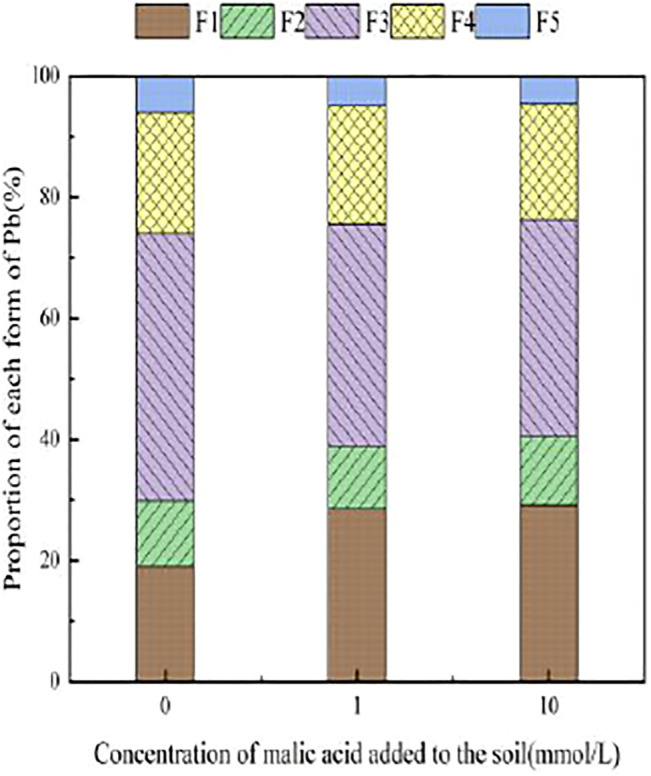
Distribution of Pb form in soil after adding malic acid and Pb-800 soil after adding malic acid.

With the addition of different concentrations of malic acid, the changes in the form of Pb in contaminated soil are shown in Figures 8–11. It can be seen that in Pb-contaminated soil, the addition of malic acid plumbum resulted in a sharp increase in exchangeable Pb. For example, in 400 and 800 mg/kg Pb-contaminated soil, the exchangeable Pb fraction increased from 17.22 and 18.96% to 34.66 and 29.12%, exhibiting an increase of 101.28 and 53.59%, respectively. Compared with that because of the addition of citric acid, the increase in exchangeable Pb because of the addition of malic acid was slightly lower, as was the overall activation effect. Carbonate-bound Pb exhibited an increasing trend from 10.34 to 15.99% in soil polluted with 100 mg/kg Pb, which was the largest increase induced by malic acid (54.64%). The growth rate of citric acid under the same conditions was 74.56%. The results of comparison indicate that citric acid activation of Pb was more effective than that of malic acid, which is consistent with the results of Qian et al. (2011). The iron–manganese oxidation state Pb fraction in contaminated soil gradually decreased with increasing malic acid concentrations, exhibiting a reduction from 42.02% in the original soil to 29.21% in the 400 mg/kg Pb sample, which was the maximum observed reduction of 67.82%. Previous research by Zheng and Zhu (2009) demonstrated that the addition of chelating agents such as EDTA, EDDS, and organic acids to soil can reduce the fraction of iron–manganese oxidation state Pb, increase the activity of Pb, and promote its conversion to exchangeable Pb. A study by Liu M.L. et al. (2018) also pointed out that high concentrations of organic acids promoted the desorption of Pb from soil. Except for the 200 mg/kg Pb treatment, the fraction of organically bound Pb increased slightly with the addition of malic acid, while the proportion of other Pb fractions decreased slightly. The residual Pb fraction did not change significantly with the addition of malic acid.

### 3.4 The Influence of Organic Acids on Dynamic Changes in Pb Form in Soil

**FIGURE 12:**
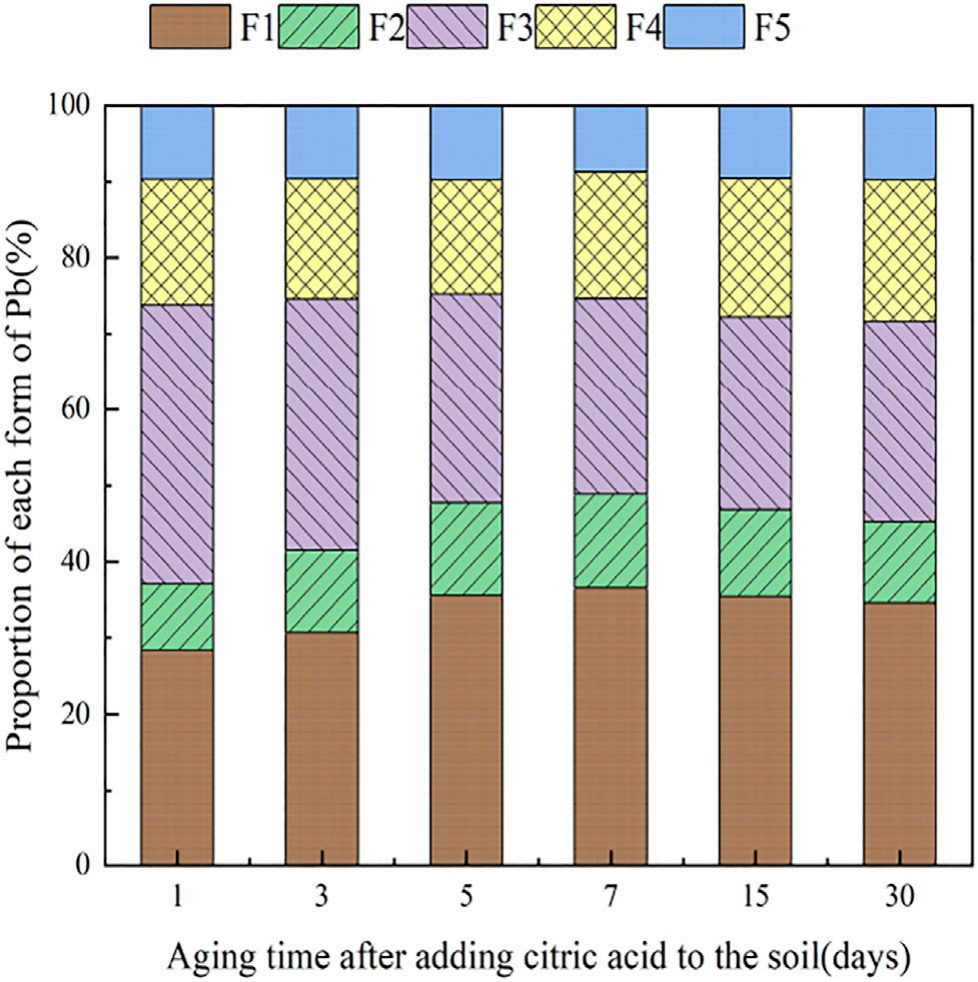
Distribution of Pb in Pb-400–contaminated soil after adding citric acid.

**FIGURE 13:**
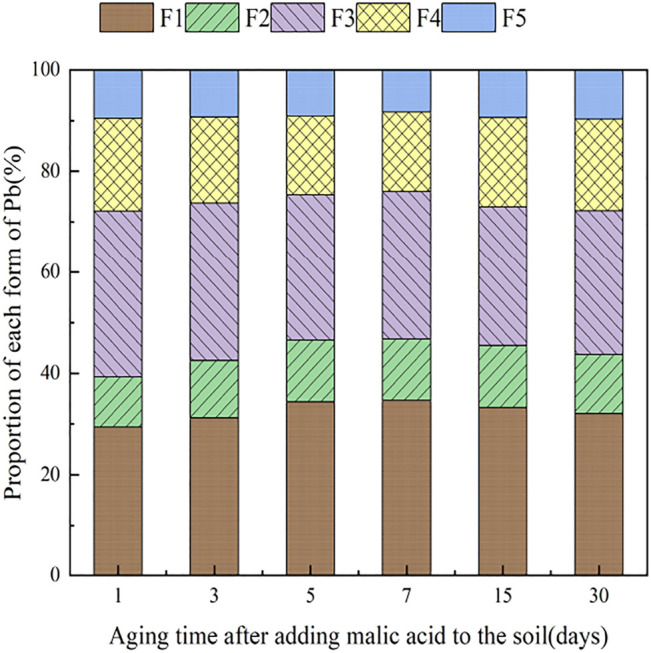
Distribution of Pb in Pb-400–contaminated soil after adding malic acid.

During the 30 days of soil incubation experiment, the change in Pb fractions in soils under the influence of the two organic acids is shown in Figures 12, 13. With the addition of citric acid and malic acid, extended incubation time resulted in an increasing trend of exchangeable Pb fraction exhibited, followed by a decrease until equilibrium is reached. With the addition of citric acid, the fraction of proportion of exchangeable Pb increased from 28.36% on the first day and reached 36.57% on the seventh day and then gradually decreased with time. By the 30th day, the fraction of exchangeable Pb decreased to 34.61%, reaching a balanced state. With the addition of malic acid, the trend in change of exchangeable Pb was similar, with the exception of that on the seventh day, as the fraction of exchangeable Pb was only 34.66%, which is lower than that of the addition of citric acid. The difference of activation effects between citric acid and malic acid occurred because the reaction between organic acids and heavy metals is related to the number and position of carboxyl and phenolic groups in the lower-molecular-weight organic acids (Liu, 2011). Citric acid is a tricarboxylic acid, while malic acid is a dicarboxylic acid, and the stability constants of citric acid and the formation of complexes are greater than those of malic acid. The solubility of organic acids for heavy metals also depends on the solubility constant of the organic acid itself, which is higher for citric acid (7.1 × 10^−4^) than for malic acid (3.9 × 10^−4^) (Fan et al., 2008), so the activation ability of citric acid for Pb is stronger than that of malic acid, which is basically consistent with the results of Zheng and Zhu (2009).

The change of the carbonate-bound state Pb presented a similar pattern to that of the exchangeable-state Pb, with a trend of increasing and then decreasing percent fraction with time. However, the change of the carbonate-bound state Pb and the exchangeable-state Pb was flat, fluctuating up and down approximately 11%, and there was little difference between the effects of citric acid and malic acid. The oxidation state of iron–manganese-bound Pb decreased significantly and reached its lowest point approximately 15 days with 25.39% (citric acid) and 27.55% (malic acid), and increased again to 26.41% (citric acid) and 28.48% (malic acid) by 30 days. The increase of corresponding values was the main contribution to the transformation of active state. The organic bound state of Pb did not change significantly during the first week of soil incubation, but slowly increased from 16.62 and 15.71% to 18.61% (citric acid) and 18.41% (malic acid) from 7 days onward. The residue state did not change significantly with the increase of incubation time. From the above trends of various forms of Pb, it is indicated that after the exogenous heavy metal Pb enters the soil medium in water-soluble form, there is an obvious aging process, i.e., the process of transformation from a more active form to a more stable form, which is basically consistent with the findings of Gleyzes et al. (2022).

## 4 Discussion

The physicochemical properties of reclaimed areas of reconstituted soils have been significantly changed. The soils were anthropogenically disturbed, mutable, and susceptible to environmental factors (Zhang et al., 2018). The addition of exogenous Pb and different types and concentrations of organic acids to the soil resulted in changes in the fraction, morphology, and biological activity of elemental Pb in the soil. The main form of Pb observed in the original reclaimed soil was residual-state Pb with less toxicity, which is chemically stable and hardly affected by environmental conditions. With the addition of exogenous Pb, the Pb in the soil changes from a residual state to an exchangeable state and a combined state with iron and manganese oxides. This reduced the relative proportion of residual Pb and increased the available fraction of active and mobile Pb in soil, which promoted the overall impact on the environment.

The adsorption–desorption process of heavy metal ions in soil media was affected by the interaction of several factors (soil composition, environmental factors, pollutant composition, etc.) (Zhang et al., 2019). Wang et al. (2017) used soil column simulation to investigate the effect of pH on the adsorption–desorption process of soil media, and the results showed that the adsorption of Pb by soil media fluctuates and increases with increasing pH within a certain range. The addition of organic acids to the soil increased the exchangeable-state Pb fraction and decreased the iron–manganese oxide-combined state in the soil, while other morphological changes were not obvious. The organic ligands dissociated from organic acids are able to interact with heavy metal ions in the soil to form stable complexes, thus altering the migration capacity of heavy metal ions (Huang, 2020). Low-molecular-weight organic acids also release more H protons to promote the desorption of heavy metals (Luo, 2016). Citric and malic acids presented similar pattern, whereas the growth rate of exchangeable Pb by adding citric acid was twice as high as that by adding malic acid (in 400 and 800 mg/kg contaminated soils), suggesting that citric acid is more beneficial in activating Pb in soils and increasing its mobility than malic acid. However, the activation effect of a single addition still needs to be improved, so it is very relevant to explore the adoption of a combination of organic acid additions. Liu (2011) added citric acid and malic acid to soil in a combined manner compared to a single addition to activate heavy metal Pb in soil more effectively because, first, the combination of two organic acids can more significantly reduce soil pH and promote the dissolution of solid-phase heavy metals in soil as the H^+^ concentration increases; second, the combination of two organic acids added reduces the amount of single organic acids chelating other competitive metal cations, converting the other more stable forms of Pb into the active exchangeable state of Pb, thus more favorable to the uptake of Pb by hyperaccumulating plants.

The topsoil capacity of the reclaimed soil was tight, and the soil nutrients were at very low levels except for quick-acting potassium and available phosphorus. Whereas organic acids are associated with specific physical and biochemical processes, the presence of organic acids changes the physical properties of the soil and may facilitate the uptake of soil nutrients. Related experiments proved (Zhao et al., 2018) that the addition of exogenous low-molecular-weight organic acids to the soil within a certain concentration range significantly increased the organic matter fraction. Zhang et al. (2019) showed that the addition of low-molecular-weight organic acids in the concentration range of 0–1 mol/L resulted in a significant increase in the available phosphorus fraction of the soil. Therefore, the addition of organic acids to reclaimed soils not only helps enhance the activity of Pb in the soil but also increases the soil organic matter fraction and promotes the uptake of soil nutrients.

## 5 Conclusion

Changes in the form of Pb in soil are affected by the addition of exogenous Pb, with continuous increases in exogenous Pb concentrations resulting in Pb existing in the form of iron–manganese oxide-combined state > organic-combined state > exchangeable state > carbonate-combined state > residual-state species. The relative proportion of residual-state Pb continuously decreased, while the fraction of iron–manganese oxide-combined Pb gradually increased, becoming the dominant form, which is beneficial for enhanced Pb activity in the soil.

The addition of citric acid and malic acid to soil increased the availability of Pb and as the concentration of organic acids increased, the degree of Pb activation increased accordingly. Citric acid gradually increased both exchangeable Pb and carbonate-binding Pb in the soil, reaching a maximum at 10 mmol/L in group C, while both exchangeable Pb and carbonate-binding Pb increased less than citric acid in the contaminated soil under the influence of malic acid under the same conditions. Comparing the two acids, we found that the overall activation effect of citric acid was stronger than that of malic acid. With extended soil aging times in the presence of citric acid and malic acid, the loosely bound Pb in soil is gradually transformed into strongly bound Pb. Therefore, citric acid, presenting a better activation effect, can be considered as a remediation aid to enhance soil remediation efficiency in the remediation of soil Pb pollution.

## Data Availability

The original contributions presented in the study are included in the article/Supplementary Material, and further inquiries can be directed to the corresponding author.
